# The rapidly convergent solutions of strongly nonlinear oscillators

**DOI:** 10.1186/s40064-016-2859-0

**Published:** 2016-08-04

**Authors:** M. S. Alam, Md. Abdur Razzak, Md. Alal Hosen, Md. Riaz Parvez

**Affiliations:** 1 Department of Mathematics, Rajshahi University of Engineering and Technology (RUET), Kazla, Rajshahi, 6204 Bangladesh; 2 Department of Mechanical Engineering, Rajshahi University of Engineering and Technology (RUET), Kazla, Rajshahi, 6204 Bangladesh

**Keywords:** Nonlinear oscillation, Harmonic balance method, Duffing equation

## Abstract

Based on the harmonic balance method (HBM), an approximate solution is determined from the integral expression (i.e., first order differential equation) of some strongly nonlinear oscillators. Usually such an approximate solution is obtained from second order differential equation. The advantage of the new approach is that the solution converges significantly faster than that obtained by the usual HBM as well as other analytical methods. By choosing some well known nonlinear oscillators, it has been verified that an *n*-th (*n* ≥ 2) approximate solution (concern of this article) is very close to (2*n* − 1)-th approximations obtained by usual HBM.

## Background

The harmonic balance method (HBM) (Mickens 1996; West 1960; Mickens 1984, 1986; Lim and Wu 2003; Lim et al. 2005; Wu et al. 2006; Belendez et al. 2006; Alam et al. 2007; Hu 2006a, b; Lai et al. 2009; Hosen et al. 2012) is a widely used technique for solving strongly nonlinear oscillators

1 $$ \textit{\"{x}} + f(x,\dot{x}) = 0,\quad [x(0) = a,\;\dot{x}(0) = 0] , $$

where $f(x,\dot{x})$ is a nonlinear function, satisfies a condition, $\,f( - x,\dot{x}) = \, - f(x,\dot{x})$. Multiplying both sides of Eq. (1) by $2\dot{x}$ and then integrating, Eq. (1) readily becomes

2 $$ \dot{x}^{2} + F(x) = F(a) , $$

where *dF*/*dx* = *f*(*x*). In general *f*(*x*) is an odd polynomial function. Therefore Eq. (2) can be written as

3 $$ G(\dot{x}^{2} ,x^{2} - a^{2} ,x^{4} - a^{4} , \ldots ) = 0 . $$

When *f*(*x*) is not a simple polynomial function (e.g., pendulum equation, $l\,\textit{\"{x}} + g\,\,\sin \,x = 0$), Eq. (3) is valid for amplitude of oscillation, *a* < 1. Sometimes the nonlinear function, *f* depends on both *x* and $\dot{x}$ (e.g., $\textit{\"{x}} + (1 + \dot{x}^{2} )x = 0$). In this case, the integral expression of such equations has been taken in the form of Eq. (3).

The modified Lindsted–Poincare method (Cheung et al. 1991; He 2002a, b; Ozis and Yildirim 2007), He’s homotopy perturbation method (Belendez et al. 2007a, b; Belendez 2007), iterative method (Mickens 1987a, b, 2005, 2010; Lim and Wu 2002; Lim et al. 2006; Hu 2006a, b; Guo et al. 2011; Haque et al. 2013), He’s energy balance method (He 2002a, b) etc. are also used to investigate nonlinear oscillators. Though, all these analytical methods (Mickens 1984, 1986, 1987a, b, 1996, 2005, 2010; West 1960; Lim and Wu 2002, 2003; Lim et al. 2005, 2006; Wu et al. 2006; Belendez et al. 2006, 2007a, b; Alam et al. 2007; Hu 2006a, b; Lai et al. 2009; Cheung et al. 1991; He 2002a, b; Ozis and Yildirim 2007; Belendez 2007; Guo et al. 2011; Haque et al. 2013) have been developed for handling nonlinear oscillator Eq. (1), they provide almost similar results for a particular approximation. Recently, HBM has been modified by truncating some higher order terms of the algebraic equations of related variables to the solution [see Hosen et al. (2012) for details] and it measures more correct result than the usual HBM solutions (derived in Wu et al. 2006; Belendez et al. 2006; Alam et al. 2007; Hu 2006a, b; Lai et al. 2009) as well as other solutions derived by several analytical methods (Belendez 2007; Belendez et al. 2007b; Mickens 1987a, b, 2005, 2010; Lim and Wu 2002; Lim et al. 2006; Hu 2006a, b; Guo et al. 2011; Haque et al. 2013; He 2002a, b). However for any approximation, the result (even the solution obtained in Hosen et al. (2012)) is not better than the next higher approximation. Moreover, the modification on HBM used in Hosen et al. (2012) is valid for some nonlinear oscillators especially when *f*(*x*) contains a term, *x*^3^. In this article, a new approach (based on the HBM) has been introduced in which the solution rapidly converges toward its exact solution. The trial solution is similar to that of Hosen et al. (2012) and the determination of the related unknowns is also similar. Yet the solution converges faster than the usual solution. Actually an *n*th (*n* ≥ 2) approximate solution of Eq. (2) is almost similar to the (2*n* − 1)-th approximation obtained from Eq. (1). To verify this statement, the second and third approximations have been obtained from the integral expressions of some important nonlinear oscillators. The new solutions are respectively close to the third and fifth approximations determined by usual HBM which are agree with the statement.

## Methods

Let us consider a periodic solution in the form (Hosen et al. 2012)

4 $$ x(t) = a\,((1 - u_{1} (a) - u_{2} (a) - \cdots )\cos \varphi (a,t) + u_{1} (a)\cos 3\varphi (a,t) + u_{2} (a)\cos 5\varphi (a,t) + \cdots ) , $$

where *a* (amplitude) and $\dot{\varphi }$ (frequency) are constants and initial phase, $\varphi$_0_(*a*) = 0. This trial solution was early used in Hosen et al. (2012) to solve Eq. (1). In this article, Eq. (4) is used for solving Eq. (2).

Differentiating *x*, squaring and simplifying, we obtain

5 $$ \begin{aligned} \dot{x}^{2} & = a_{0}^{2} \dot{\varphi }^{2} \sin^{2} \varphi \,(1 + 4u_{1} + 8u_{2} + 22u_{1}^{2} + 76u_{1} u_{2} + 116u_{2}^{2} + (12u_{1} + 20u_{2} + 24u_{1}^{2} + 148u_{1} u_{2} \\ & \quad + 180u_{2}^{2} )\cos 2\varphi + (20u_{2} + 18u_{1}^{2} + 100u_{1} u_{2} + 130u_{2}^{2} )\cos 4\varphi + \cdots ). \\ \end{aligned} $$

Then we have determined an expression for *x*^2^ − *a*^2^, as

6 $$ \begin{aligned} x^{2} - a^{2} & = - a^{2} \sin^{2} \varphi \;(1 + 4u_{1} + 8u_{2} - 2u_{1}^{2} - 4u_{1} u_{2} - 4u_{2}^{2} + (4u_{1} + 12u_{2} - 4u_{1} u_{2} \\ & \quad - 4u_{2}^{2} )\cos 2\varphi + (4u_{2} + 2u_{1}^{2} + 4u_{1} u_{2} + 2u_{2}^{2} )\cos 4\varphi + \cdots )\,. \\ \end{aligned} $$

All other expressions *x*^4^ − *a*^4^, *x*^6^ − *a*^6^, … of Eq. (3) have a factor *x*^2^ − *a*^2^; so that a common factor ${a^{2}} {\sin^{2}} \varphi$ must be cancelled when all these values are substituted in Eq. (3). It is noted that the canceling of the common (i.e., ${a^{2}} {\sin^{2}} \varphi$) factor makes the solution better than usual solution. Otherwise the solution does not converge fast. It also makes the solution different from that obtained by the energy balance method.

## Examples

### Quintic Duffing oscillator

Let us consider quintic Duffing oscillator, i.e.,

7 $$ \textit{\"{x}} + x + x^{5} = 0 . $$

By utilization of initial conditions, $[x(0) = a,\;\dot{x}(0) = 0]$, Eq. (7) readily takes the form

8 $$ \dot{x}^{2} + (x^{2} - a^{2} ) + (x^{6} - a^{6} )/3 = 0 . $$

It has already been mentioned that an analytical solution can be obtained either from Eq. (7) or from Eq. (8). The aim of this article is to find approximate solution from Eq. (8) rather than Eq. (7). A third approximate solution (in which *u*_1_ and *u*_2_ are non-zero) has been mainly considered. Sometimes a second approximate solution has been considered to compare it with existing solution obtained by several authors.

Substituting solution Eq. (4) (together with *u*_*j*_ = 0, *j* > 2) in Eq. (8), dividing by ${a^{2}} {\sin^{2}} \varphi$ and equating the coefficient of Constant, cos 2$\varphi$ and cos 4$\varphi$, the following nonlinear algebraic equations are obtained

9 $$ \begin{aligned} & \dot{\varphi }^{2} (1 + 4u_{1} + 8u_{2} + 22u_{1}^{2} + 76u_{1} u_{2} + 116u_{2}^{2} ) - (1 + 4u_{1} + 8u_{2} - 2u_{1}^{2} - 4u_{1} u_{2} - 4u_{2}^{2} ) - 5a^{4} (1 + 4u_{1} \\ & \quad + 10u_{2} - 5u_{1}^{2} - 18u_{1} u_{2} - 19u_{2}^{2} + 8u_{1}^{3} - 10u_{1}^{4} + 32u_{1}^{2} u_{2} + 56u_{1} u_{2}^{2} + 36u_{2}^{3} - 40u_{1}^{3} u_{2} + \cdots )/8 = 0, \\ & \dot{\varphi }^{2} (12u_{1} + 20u_{2} + 24u_{1}^{2} + 148u_{1} u_{2} + 180u_{2}^{2} ) - (4u_{1} + 12u_{2} - 4u_{1} u_{2} - 4u_{2}^{2} ) \\ & \quad - a^{4} (4 + 45u_{1} + 123u_{2} - 30u_{1}^{2} - 150u_{1} u_{2} - 180u_{2}^{2} + 20u_{1}^{3} + \cdots )/12 = 0, \\ & \dot{\varphi }^{2} (20u_{2} + 18u_{1}^{2} + 100u_{1} u_{2} + 130u_{2}^{2} ) - (4u_{2} + 2u_{1}^{2} + 4u_{1} u_{2} + 2u_{2}^{2} ) \\ & \quad - a^{4} (1 + 36u_{1} + 132u_{2} + 60u_{1}^{2} + 120u_{1} u_{2} - 160u_{1}^{3} + 225u_{1}^{4} + \cdots )/24 = 0. \\ \end{aligned} $$

By elimination of $\dot{\varphi }$ from three equations of Eq. (9), we obtain two equations of *u*_1_ and *u*_2_ as

10 $$ \begin{aligned} & 24u_{1} + 24u_{2} + 456u_{1} u_{2} + 72u_{1}^{2} - 864u_{1}^{3} - 4320u_{1}^{2} u_{2} + 552u_{2}^{2} - 9120u_{1} u_{2}^{2} - 7200u_{2}^{3} + a^{4} ( - 4 + 45u_{1} \\ & \quad + 27u_{2} + 1440u_{1} u_{2} + 210u_{1}^{2} - 2450u_{1}^{3} - 13050u_{1}^{2} u_{2} + 1830u_{2}^{2} - 27060u_{1} u_{2}^{2} - 19950u_{2}^{3} )/4 = 0, \\ & 384u_{1}^{2} + 384u_{2} + 2304u_{1} u_{2} + 3072u_{2}^{2} - 11520u_{1}^{2} u_{2} - 38400u_{1} u_{2}^{2} - 57600u_{2}^{3} + a^{4} ( - 1 - 36u_{1} + 168u_{2} \\ & \quad + 210u_{1}^{2} + 1380u_{1} u_{2} - 160u_{1}^{3} + 2505u_{2}^{2} - 7020u_{1}^{2} u_{2} - 26880u_{1} u_{2}^{2} - 41060u_{2}^{3} ) = 0. \\ \end{aligned} $$

In general, *u*_1_ and *u*_2_ are small. So, it is possible to divide the first and second of Eq. (10) respectively by $1 + 3u_{1} + 19u_{2} - 36u_{1}^{2} - 180u_{1} u_{2} - 380u_{2}^{2}$ and $1 + 6u_{1} + 8u_{2} - 30u_{1}^{2} - 100u_{1} u_{2} - 150u_{2}^{2}$, and then they become

11 $$ \begin{aligned} & - 24u_{1} - 24u_{2} + 72u_{1} u_{2} - 1080u_{1}^{2} u_{2} - 96u_{2}^{2} - 5400u_{1} u_{2}^{2} - 96u_{2}^{3} \\ & \quad + a^{4} \left( {4 - 57u_{1} - 103u_{2} + 672u_{1} u_{2} + 105u_{1}^{2} + 83u_{1}^{3} - 4929u_{1}^{2} u_{2} /2 + 1647u_{2}^{2} } \right)/4 = 0, \\ & - 384u_{1}^{2} - 384u_{2} + 2304u_{2}^{3} + 3072u_{1}^{2} u_{2} + a^{4} (1 + 30u_{1} - 176u_{2} - 360u_{1}^{2} - 464u_{1} u_{2} - 947u_{2}^{2} ) = 0 \\ \end{aligned} $$

Now, the above equations can written as

12 $$ \begin{aligned} u_{1} & = \lambda \left( {1 - \frac{{103u_{2} }}{4} + 168u_{1} u_{2} + \frac{{105u_{1}^{2} }}{4} + \frac{{83u_{1}^{3} }}{4} - \frac{{4929u_{1}^{2} u_{2} }}{4} + \frac{{1647u_{2}^{2} }}{4}} \right) + \left( {\frac{1}{24} - \frac{19\lambda }{32}} \right) \\ & \quad \times \left( { - 24u_{2} + 72u_{1} u_{2} - 1080u_{1}^{2} u_{2} - 96u_{2}^{2} - 5400u_{1} u_{2}^{2} - 96u_{2}^{3} } \right), \\ u_{2} & = \left( {\frac{\lambda }{16} + \frac{{13\lambda^{2} }}{64} + \frac{{169\lambda^{2} }}{256}} \right)\left( {1 + 30u_{1} - 360u_{1}^{2} - 464u_{1} u_{2} - 947u_{2}^{2} } \right) + \left( {\frac{1}{384} - \frac{11\lambda }{384} + \frac{{143\lambda^{2} }}{1536}} \right) \\ & \quad \times \left( { - 384u_{1}^{2} + 2304u_{2}^{3} + 3072u_{1}^{2} u_{2} } \right), \\ \end{aligned} $$

where *λ* = 4*a*^4^/(96 + 57*a*^4^). It is clear that *λ* is much smaller than 1 for every values of *a*. As *a* → ∞, *λ* becomes 4/57 (which is the largest). Therefore, *u*_1_ and *u*_2_ can be obtained in powers of *λ* of the forms *u*_1_ = *l*_1_*λ* + *l*_2_*λ*^2^ + ··· and *u*_2_ = *m*_1_*λ* + *m*_2_*λ*^2^ + ··· [see Hosen et al. (2012) for details]. Substituting the series of *u*_1_ and *u*_2_ in Eq. (12) and equating the equal powers of *λ*, a set of linear algebraic equations of *l*_1_, *l*_2_, …, *m*_1_, *m*_2_, …, are obtained. Solving these algebraic equations, the unknown constants, *l*_1_, *l*_2_, …, *m*_1_, *m*_2_, … are determined. Thus *u*_1_ and *u*_2_ become

13 $$ \begin{aligned} u_{1} & = \frac{15\lambda }{16} - \frac{{105\lambda^{2} }}{64} + \frac{{38865\lambda^{3} }}{2048} - \cdots , \\ u_{2} & = \frac{\lambda }{16} + \frac{{277\lambda^{2} }}{256} - \frac{{1153\lambda^{3} }}{4096} - \cdots . \\ \end{aligned} $$

Now substituting these values of *u*_1_ and *u*_2_ in the first equation of Eq. (9) and then simplifying, the frequency (i.e., $\dot{\varphi }$) is obtained. It is noted that the series of *u*_1_ and *u*_2_ are valid for all values of *a*. For some particular values of *a*, the approximate frequency has been calculated and presented in Table 1. When *a* < 1, this approximate solution can be compare with some results obtained by usual HBM. In this case, *λ* can be expanded in powers of *a* and the series of $\dot{\varphi }^{2}$ becomes

14 $$ \dot{\varphi }_{3(q)}^{2} = 1 + \frac{{5a^{4} }}{8} - \frac{{65a^{8} }}{1536} + \frac{{1055a^{12} }}{36864} - \frac{{129906\tfrac{7}{48}a^{16} }}{6291456} + \cdots . $$

**Table 1:** Comparison the approximate frequencies of Eq. (7) between the present method and the usual HBM method with the exact frequency $\dot{\varphi }_{Ex}$, obtained by direct numerical integration

*a*	$\dot{\varphi }_{Ex}$	$\dot{\varphi }_{3(q,Usual)}$ *Er* (%)	$\dot{\varphi }_{3(q,Present)}$ *Er* (%)
0.5	1.0192663	1.0192661	1.0192663
		0.00002	0.00000
0.7	1.0714295	1.0714202	1.0714295
		0.00087	0.00000
1	1.26471	1.26446	1.26469
		0.020	0.002
2	3.16666	3.16223	3.16639
		0.140	0.008
3	6.80379	6.79391	6.80382
		0.145	0.000
4	11.9959	11.9785	11.9963
		0.145	0.003
5	18.7007	18.6736	18.7014
		0.145	0.003
10	74.6909	74.5829	74.6941
		0.145	0.004
50	1867.09	1864.39	1867.17
		0.145	0.004
100	7468.34	7457.55	7468.66
		0.145	0.004

*Er* (%) denotes absolute percentage error

The exact value of $\dot{\varphi }^{2}$ is

15 $$ \dot{\varphi }_{Ex(q)}^{2} = 1 + \frac{{5a^{4} }}{8} - \frac{{65a^{8} }}{1536} + \frac{{1055a^{12} }}{36864} - \frac{{129545a^{16} }}{6291456} + \cdots , $$

where $\dot{\varphi }_{3(q)}^{2}$ and $\dot{\varphi }_{Ex(q)}^{2}$ denote respectively, the third approximate frequency by the present method and exact frequency of the Eq. (7).

Comparing Eqs. (14) and (15), it is clear that the first four terms of $\dot{\varphi }_{3(q)}^{2}$ obtained in Eq. (14) are identical to those of its exact result, $\dot{\varphi }_{Ex(q)}^{2}$. But the result of $\dot{\varphi }_{3(q)}^{2}$ is different from that of $\dot{\varphi }_{3(q,Usual)}^{2}$ obtained by the usual HBM [see Eq. (39) of Appendix 1: though the solution is obtained from Eq. (7) containing two higher harmonic terms *u*_1_ and *u*_2_]. We see that first three terms of $\dot{\varphi }_{3(q,Usual)}^{2}$ are identical to its exact result. It is noted that the first four terms of $\dot{\varphi }_{3(q,Usual)}^{2}$ would be same those of $\dot{\varphi }_{Ex(q)}^{2}$, when the solution is derived from Eq. (7) containing four higher harmonic terms *u*_1_, *u*_2_, *u*_3_ and *u*_4_. Certainly, it is a laborious task to determine five unknown *u*_1_, *u*_2_, *u*_3_, *u*_4_ and $\dot{\varphi }^{2}$ for any analytical method.

### Cubic Duffing oscillator

Let us consider cubic Duffing oscillator, i.e.,

16 $$ \textit{\"{x}} + x + x^{3} = 0 . $$

By utilization of initial conditions, $[x(0) = a,\;\dot{x}(0) = 0]$, Eq. (16) readily takes the form

17 $$ \dot{x}^{2} + (x^{2} - a^{2} ) + (x^{4} - a^{4} )/2 = 0 . $$

First of all we consider a third approximate solution in which *u*_1_ and *u*_2_ are non-zero. Substituting solution Eq. (4) in Eq. (17), dividing by ${a^{2}} {\sin^{2}} \varphi$ and equating the coefficient of Constant, cos 2$\varphi$ and cos 4$\varphi$, we obtain

18 $$ \begin{aligned} & \dot{\varphi }^{2} (1 + 4u_{1} + 8u_{2} + 22u_{1}^{2} + 76u_{1} u_{2} + 116u_{2}^{2} ) - (1 + 4u_{1} + 8u_{2} - 2u_{1}^{2} - 4u_{1} u_{2} - 4u_{2}^{2} ) - 3a^{2} (1 \\ & \quad + 4u_{1} + 28u_{2} /3 - 12u_{1} u_{2} - 4u_{1}^{2} - 12u_{2}^{2} + 4u_{1}^{3} - 2u_{1}^{4} + 12u_{1}^{2} u_{2} - 16u_{1}^{3} u_{2} /3 + \cdots )/4 = 0, \\ & \dot{\varphi }^{2} (12u_{1} + 20u_{2} + 24u_{1}^{2} + 148u_{1} u_{2} + 180u_{2}^{2} ) - 4(u_{1} + 3u_{2} - u_{1} u_{2} - u_{2}^{2} ) - a^{2} (1 + 16u_{1} \\ & \quad - 6u_{1}^{2} + 2u_{1}^{4} + 44u_{2} - 36u_{1} u_{2} + 24u_{1}^{2} u_{2} - 8u_{1}^{3} u_{2} - 42u_{2}^{2} )/4 = 0, \\ & \dot{\varphi }^{2} (20u_{2} + 18u_{1}^{2} + 100u_{1} u_{2} + 130u_{2}^{2} ) - 2(2u_{2} + u_{1}^{2} + 2u_{1} u_{2} + u_{2}^{2} ) \\ & \quad - a^{2} (u_{1} + 3u_{1}^{2} - 4u_{1}^{3} + 2u_{1}^{4} + 5u_{2} + 6u_{1} u_{2} - 12u_{1}^{2} u_{2} + 7u_{1}^{3} u_{2} + 3u_{2}^{2} ) = 0. \\ \end{aligned} $$

By elimination of $\dot{\varphi }$ from three equations of Eq. (18) and then simplifying (discussed in “Quintic Duffing oscillator” section), the following relations of *u*_1_ and *u*_2_ are obtained as

19 $$ \begin{aligned} u_{1} & = \mu (1 - 35u_{2} + 27u_{1}^{2} + 194u_{1} u_{2} + 3u_{2}^{2} - 45u_{1}^{3} - 1971u_{1}^{2} u_{2} + 245u_{1}^{4} + 15232u_{1}^{3} u_{2} - 5451u_{1}^{5} ) \\ & \quad + (1 - 23\mu )( - u_{2} + 3u_{1} u_{2} - 4u_{2}^{2} - 45u_{1}^{2} u_{2} + 315u_{1}^{3} u_{2} ), \\ u_{2} & = (2\mu + 6\mu^{2} + 18\mu^{3} + 54\mu^{4} + 162\mu^{5} + 486\mu^{6} + 1458\mu^{7} )(u_{1} - 33u_{1}^{2} /2 - 17u_{1} u_{2} \\ & \quad - 69u_{2}^{2} /2 + 125u_{1}^{3} + 294u_{1}^{2} u_{2} - 772u_{1}^{4} - 2877u_{1}^{3} u_{2} + 3588u_{1}^{5} ) + (1 - 20\mu - 60\mu^{2} - 180\mu^{3} \\ & \quad - 540\mu^{4} - 1620\mu^{5} - 4860\mu^{6} )( - u_{1}^{2} + 6u_{1}^{3} + 8u_{1}^{2} u_{2} - 39u_{1}^{4} - 96u_{1}^{3} u_{2} + 186u_{1}^{5} ), \\ \end{aligned} $$

where *μ* = *a*^2^/(32 + 23*a*^2^). It is clear that *μ* is much smaller than 1 for every values of *a*. As *a* → ∞, *μ* becomes 1/23 (which is the largest). For every values of *a*, *u*_1_ and *u*_2_ can be express in terms of *μ* (as discuss in “Quintic Duffing oscillator” section) and that are calculated as

20 $$ \begin{aligned} u_{1} & = \mu - \mu^{2} + 19\mu^{3} - 62\mu^{4} + 670\mu^{5} + 1288\mu^{6} \, + \, 18981\mu^{7} + \, 384658 \, \mu^{8} + \cdots , \\ u_{2} & = \mu^{2} - \mu^{3} + 45\mu^{4} - 215\mu^{5} + 1004\mu^{6} - 13589 \, \mu^{7} + \, 7668\mu^{8} + \, \cdots . \\ \end{aligned} $$

Substituting the values of *u*_1_ and *u*_2_ into the first equation Eq. (18), and then simplifying, it becomes

21 $$ \dot{\varphi }_{3(c)}^{2} = 1 + \frac{{3a^{2} }}{4} - \frac{{3a^{4} }}{128} + \frac{{9a^{6} }}{512} - \frac{{1779a^{8} }}{131072} + \frac{{5643a^{10} }}{524288} - \frac{{146542\tfrac{1}{8}a^{12} }}{16777216} + \cdots . $$

The exact value of $\dot{\varphi }^{2}$ is

22 $$ \dot{\varphi }_{Ex(c)}^{2} = 1 + \frac{{3a^{2} }}{4} - \frac{{3a^{4} }}{128} + \frac{{9a^{6} }}{512} - \frac{{1779a^{8} }}{131072} + \frac{{5643a^{10} }}{524288} - \frac{{146661a^{12} }}{16777216} + \cdots , $$

where $\dot{\varphi }_{3(c)}^{2}$ and $\dot{\varphi }_{Ex(c)}^{2}$ denote respectively, the third approximate frequency by the present method and exact frequency of the Eq. (16).

We see that first six terms of Eq. (21) are identical to the exact result in Eq. (22), and error occurs slightly in 7th term. It is noted that only the four terms of Eq. (45) [see “Appendix  2” and also (7–10)] are identical to the exact frequency when a third approximate solution is obtained from original equation Eq. (16). On the contrary, six terms of the fifth approximate solution (obtained by usual HBM) would be identical to its exact result $\dot{\varphi }_{Ex(c)}^{2}$. It has already been mentioned that the derivation of a fifth approximate solution is very laborious.

### A strongly nonlinear oscillator containing $\dot{x}^{2}$

Now we consider the nonlinear oscillator

23 $$ \textit{\"{x}} + (1 + \dot{x}^{2} )x = 0 . $$

By introducing a scaling variable *ɛ*, as $x(t) = \sqrt \varepsilon \,y(t)$, 0 < *ɛ* < 1, Eq. (23) can be easily transformed to a weak nonlinear equation, $\textit{\"{y}} + y + \varepsilon \,y\,\dot{y}^{2} = 0$ and it has a perturbation solution [see Belendez et al. (2007c) for details]. The aim of this article is to obtain another approximate solution. An integral expression of this equation is

$$ \ln (1 + \dot{x}^{2} ) + x^{2} = a^{2} $$

or,

24 $$ \dot{x}^{2} = \exp (a^{2} - x^{2} ) - 1 $$

When *a* ≤ 1, exp (*a*^2^ − *x*^2^) can be expanded in the Maclaurin series and Eq. (24) becomes

25 $$ \dot{x}^{2} + (x^{2} - a^{2} ) - \frac{{(x^{2} - a^{2} )^{2} }}{2} + \frac{{(x^{2} - a^{2} )^{3} }}{6} - \frac{{(x^{2} - a^{2} )^{4} }}{24} + \frac{{(x^{2} - a^{2} )^{5} }}{120} - \cdots = 0 . $$

Substituting solution Eq. (4) in Eq. (25), dividing by ${a^{2}} {\sin^{2}} \varphi$ and equating the coefficient of Constant, cos 2$\varphi$, we obtain

26 $$ \begin{aligned} & \dot{\varphi }^{2} (1 + 4u_{1} + 22u_{1}^{2} ) - (1 + 4u_{1} - 2u_{1}^{2} ) - a^{2} (1 + 4u_{1} + 4u_{1}^{2} + \cdots )/4 \\ & \quad - a^{4} (1 + 4u_{1} + 7u_{1}^{2} + \cdots )/16 - a^{6} (5 + 20u_{1} + 44u_{1}^{2} + \cdots )/384 + \cdots = 0, \\ & \dot{\varphi }^{2} (12u_{1} + 24u_{1}^{2} ) - 4u_{1} + a^{2} (1 - 6u_{1}^{2} )/4 + a^{4} (2 + 3u_{1} - 6u_{1}^{2} + \cdots )/24 \\ & \quad + a^{6} (147 + 1568u_{1} - 392u_{1}^{2} + \cdots )/ 3 7 6 3 2+ \cdots = 0. \\ \end{aligned} $$

By elimination of $\dot{\varphi }$ from two equations of Eq. (26), the equation of *u*_1_ is obtained as

27 $$ a^{2} + a^{4} /3 + 5a^{6} /64 + 32u_{1} + 16a^{2} u_{1} + 29a^{4} u_{1} /6 + 224u_{1}^{2} + 88a^{2} u_{1}^{2} - 64u_{1}^{3} = 0. $$

The coefficient of *u*_1_ is much greater than the coefficients of *a*^2^, *a*^4^, *a*^6^, ···, so Eq. (27) can be solved by choosing *u*_1_ = *l*_1_*a*^2^ + *l*_2_*a*^4^ + *l*_3_*a*^6^ + ···, where the unknown coefficients, *l*_1_, *l*_2_, *l*_3_, …, to be determined. Doing all these, the solution becomes

28 $$ u_{1} = - \frac{{a^{2} }}{32} - \frac{{5a^{4} }}{3072} - \frac{{3a^{6} }}{8192} - \cdots . $$

Now substituting the value of *u*_1_ in the first equation Eq. (26), the approximate frequency (i.e., $\dot{\varphi }$) for small oscillation is obtained as

29 $$ \dot{\varphi }_{2}^{2} = 1 + \frac{{a^{2} }}{4} + \frac{{5a^{4} }}{128} + \frac{{5a^{6} }}{1536} - \cdots , $$

The exact value of $\dot{\varphi }^{2}$ is

30 $$ \dot{\varphi }_{Ex}^{2} = 1 + \frac{{a^{2} }}{4} + \frac{{5a^{4} }}{128} + \frac{{5a^{6} }}{1536} - \frac{{3a^{8} }}{131072} - \frac{{91a^{10} }}{ 2 6 2 1 4 4 0} - \frac{{293a^{12} }}{150994944} \cdots . $$

In a similar way, the third approximate solution of Eq. (23) can be obtained as

31 $$ \begin{aligned} u_{1} & = - \frac{{a^{2} }}{32} - \frac{{a^{4} }}{256} + \frac{{a^{6} }}{16384} + \cdots , \\ u_{2} & = \frac{{7a^{4} }}{3072} + \frac{{7a^{6} }}{32768} + \cdots , \\ \end{aligned} $$

and

32 $$ \dot{\varphi }_{3}^{2} = \,1 + \frac{{a^{2} }}{4} + \frac{{5a^{4} }}{128} + \frac{{5a^{6} }}{1536} - \frac{{3a^{8} }}{131072} - \frac{{91a^{10} }}{ 2 6 2 1 4 4 0} - \cdots . $$

Comparing Eqs. (29) and (32) to Eq. (30), it is clear that second and third approximations respectively measure four and six terms in correct figures. On the contrary, the usual HBM is able to respectively measure three and four terms in correct figures (see “Appendix 3”). Thus the statement is true for nonlinear oscillator, Eq. (23) [or, Eq. (24)].

It is noted that the series given in Eq. (31) is converge only for the small amplitudes in the region *a* ≤ 1.

## Results and discussions

A new analytical approach based on the HBM has been presented to obtain approximate solutions of some well known nonlinear oscillators. Usually, a harmonic balance solution is obtained from the second order equations. Earlier, He (2002a, b) obtained some approximate solutions (mainly first approximation) for various nonlinear oscillators from corresponding first order differential equations (i.e., energy balance equations). But the new approach (concern of this article) is entirely different from He (2002a, b) technique. In this article, the first order equation is rewritten in such a way that every term is completely divisible by ${a^{2}} {\sin^{2}} \varphi$ for the proposed solution Eq. (4) (see “Methods” section). For three well known nonlinear problems, it has been verified that the solutions are better than corresponding solutions obtained by usual HBM. Recently, Hosen et al. (2012) have developed a technique based on the same method (i.e., HBM), but their solutions are significantly improved for the quadratic and cubic Duffing oscillators (see Hosen et al. (2012) details). On the contrary, the solution obtained by the new approach is better than usual harmonic solution even for the quintic Duffing oscillator.

**Fig. 1:**
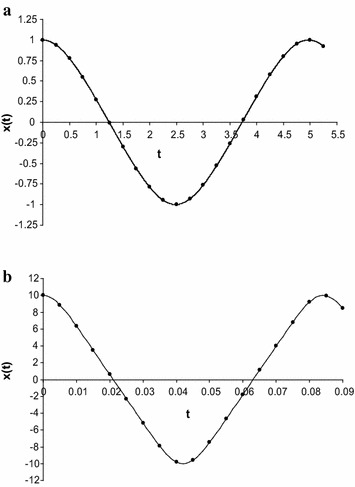
**a** Comparison of approximate periodic solution of Eq. (7) obtained by present method (denoting by *circles*) with numerical solution obtained by fourth order Runge–Kutta method (denoted by *solid line*) for *A* = 1. **b** Comparison of approximate periodic solution of Eq. (7) obtained by present method (denoting by *circles*) with numerical solution obtained by fourth order Runge–Kutta method (denoted by *solid line*) for *A* = 10

**Fig. 2:**
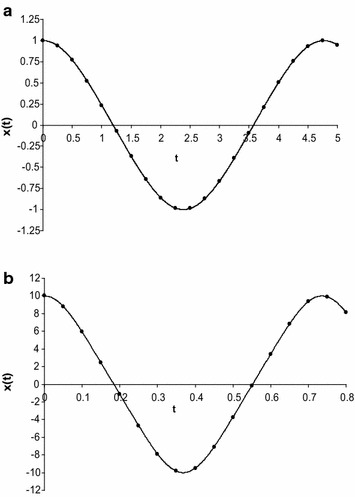
**a** Comparison of approximate periodic solution of Eq. (16) obtained by present method (denoting by *circles*) with numerical solution obtained by fourth order Runge–Kutta method (denoted by *solid line*) for *A* = 1. **b** Comparison of approximate periodic solution of Eq. (16) obtained by present method (denoting by *circles*) with numerical solution obtained by fourth order Runge–Kutta method (denoted by *solid line*) for *A* = 10

**Fig. 3:**
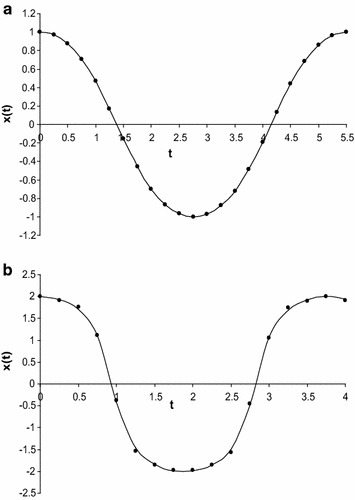
**a** Comparison of approximate periodic solution of Eq. (23) obtained by present method (denoting by *circles*) with numerical solution obtained by fourth order Runge–Kutta method (denoted by *solid line*) for *A* = 1. Comparison of approximate periodic solution of Eq. (23) obtained by present method (denoting by *circles*) with numerical solution obtained by fourth order Runge–Kutta method (denoted by *solid line*) for *A* = 2

To check the results, we have calculated the approximate frequency of Eq. (7) for some particular values of *a* (both small and large) by using Eq. (13) into the first Eq. (9) and compared with numerical solution together with other existing solutions (those solutions obtained by Wu et al. 2006; Belendez et al. 2006; Alam et al. 2007; Hu 2006a, b; Lai et al. 2009; Hosen et al. 2012) (see also “Appendix 1”) and which is presented in Table 1. The Table 1 indicates that the approximate frequencies obtained by new approach are better than those obtained by usual HBM. Next, for some particular values of *a* (both small and large), we have calculated the approximate frequency of Eq. (16) by using the Eq. (20) into the first Eq. (18) and compared with numerical solution together with other existing solutions and which is presented in Table 2. The Table 2 indicates that the approximate frequencies give good agreement with the corresponding numerical result and also give better result than those obtained by the other usual HBM. Finally, for some particular values of *a*, we have also calculated the approximate frequency of Eq. (23) by using the Eq. (31) into the first Eq. (26) and compared with numerical solution together with other existing solutions obtained by usual HBM and which is presented in Table 3. The Table 3 indicates that the approximate frequencies give better result than those obtained by the other usual HBM. Moreover, we have determined the approximate periodic solution of Eqs. (7), (16), and (23) for different values of *A* and those solutions have been presented in Figs. 1a, b, 2a, b, 3a, b. All figures have been included the corresponding numerical solutions obtained by fourth-order Runge–Kutta method.

**Table 2:** Comparison the approximate frequencies of Eq. (16) between the present method and truncation HBM Hosen et al. (2012), the usual HBM method with the exact frequency $\dot{\varphi }_{Ex}$, obtained by direct numerical integration

*a*	$\dot{\varphi }_{Ex}$	$\dot{\varphi }_{3(c,Usual(trunc))}$ (Hosen et al. 2012) *Er* (%)	$\dot{\varphi }_{3(c,Usual)}$ *Er* (%)	$\dot{\varphi }_{3(c,Present)}$ *Er* (%)
0.5	1.0891582	1.0891582	1.0891582	1.0891582
		0.00000	0.00000	0.00000
0.7	1.1676370	1.1676370	1.1676374	1.1676370
		0.00000	0.00004	0.00000
1	1.31778	1.31778	1.31778	1.31778
		0.000	0.000	0.000
2	1.97602	1.97601	1.97607	1.97602
		0.000	0.003	0.000
3	2.73849	2.73847	2.73862	2.73849
		0.000	0.005	0.000
4	3.53924	3.53921	3.53946	3.53926
		0.000	0.006	0.000
5	4.35746	4.35741	4.35777	4.35748
		0.001	0.007	0.000
10	8.53359	8.53347	8.5343	8.53363
		0.002	0.008	0.000
50	42.3730	42.3724	42.3767	42.3732
		0.002	0.009	0.000
100	84.7275	84.7262	84.7349	84.7279
		0.002	0.009	0.000

*Er* (%) denotes absolute percentage error

**Table 3:** Comparison the approximate frequencies of Eq. (23) between the present method and the usual HBM method with the exact frequency $\dot{\varphi }_{Ex}$, obtained by direct numerical integration

*a*	$\dot{\varphi }_{Ex}$	$\dot{\varphi }_{3(q,Usual)}$ *Er* (%)	$\dot{\varphi }_{3(q,\Pr esent)}$ *Er* (%)
1.8	1.52154	1.52669	1.52180
		0.339	0.017
2.0	1.67047	1.68325	1.67091
		0.765	0.026
2.2	1.84092	1.86926	1.84103
		1.540	0.006
2.4	2.03064	2.0876	2.028
		2.805	0.130
2.6	2.236	2.34108	2.22324
		4.700	0.571
2.8	2.45251	2.63242	2.41143
		7.336	1.675

From these six figures, we see that the present method provides good agreement with the corresponding numerical solution.

Furthermore, the first-order approximate frequency obtained by usual harmonic balance method (HBM) is

33 $$ \dot{\varphi } = \frac{2}{{\sqrt {4 - a^{2} } }} $$

From Eq. (33), it is observed that the approximate frequency, $\dot{\varphi }$ is undefined at *a* = 2. It is a big shortcoming of usual HBM.

On the other hand, the first-order approximate frequency becomes

34 $$ \dot{\varphi } = \sqrt {e^{{a^{2} /2}} \left( {J_{0} (a^{2} /2) - J_{1} (a^{2} /2)} \right)} , $$

according to the present method. Here *J*_0_ and *J*_1_ are Bessell’s functions. From Eq. (34), it is clear that the first approximate frequency is finite for all values of *a*. However, the relative error gradually increases as the amplitude increases.

It has already been mentioned that the series Eq. (31) is mainly converged in the region *a* ≤ 1, but the series also gives significant better result for obtaining approximate frequency even the amplitude increases up to *a* = 2.8 (see Table 3). On the contrary, the solution is only valid for in the region *a* ≤ 1 while the amplitude increases, the solutions are deviated from the numerical solution (see Fig. 3b). Comparing the approximate frequency obtained by usual HBM with the exact approximate frequency determined numerically, it is shown from Table 3 that the relative error of the approximate value is less than 5, 8 % for *a* < 2.6 and *a* < 2.8, respectively while the relative error of the approximate frequency obtained in present method is less than 0.6, 2 % for *a* < 2.6 and *a* < 2.8, respectively. Therefore, the present method is faster than usual HBM.

## Conclusion

Based on HBM, a new technique has been presented for solving a class of nonlinear oscillators. In the case of small values of amplitude, it has been verified that the fourth-order approximate frequency obtained by usual HBM is almost same as the third-order approximate frequency obtained by new method. For the case of large values of amplitude, the approximate frequencies obtained by new method not only gives better results than usual HBM but also gives nicely close to their exact results. Therefore, the results obtained in this paper are much better than those obtained by the usual HBM. The method also proved that it is a powerful mathematical tool for solving nonlinear oscillators.

## Appendix 1

Substituting solution Eq. (4) (together with *u*_*j*_ = 0, *j* > 2) in Eq. (7) and equating the coefficient of cos $\varphi$, cos 3$\varphi$ and cos 5$\varphi$, the following nonlinear algebraic equations are obtained

35 $$ \begin{aligned} & \dot{\varphi }^{2} ( - 1 + u_{1} + u_{2} ) + (1 - u_{1} - u_{2} ) + 5a^{4} (2 - 5u_{1} - 9u_{2} + 12u_{1}^{2} + 32u_{1} u_{2} + 28u_{2}^{2} - 20u_{1}^{3} + 20u_{1}^{4} \\ & \quad - 60u_{1}^{2} u_{2} + 60u_{1}^{3} u_{2} - 146u_{1} u_{2}^{2} - 50u_{2}^{3} + \cdots )/16 = 0, \\ & - 9u_{1} \dot{\varphi }^{2} + u_{1} + 5a^{4} (1 + u_{1} - u_{2} - 8u_{1}^{2} - 8u_{1} u_{2} - 2u_{2}^{2} + 20u_{1}^{3} - 25u_{1}^{4} + 30u_{1}^{2} u_{2} + 30u_{1} u_{2}^{2} - 40u_{1}^{3} u_{2} \\ & \quad + 8u_{2}^{3} + \cdots )/16 = 0, \\ & - 25u_{2} \dot{\varphi }^{2} + u_{2} + 5a^{4} (5 + 3u_{1} + 5u_{2} - 8u_{1}^{2} - 28u_{1} u_{2} - 22u_{2}^{2} + 10u_{1}^{3} - 5u_{1}^{4} + 60u_{1}^{2} u_{2} + 84u_{1} u_{2}^{2} - 64u_{1}^{3} u_{2} \\ & \quad + 46u_{2}^{3} + \cdots )/16 = 0. \\ \end{aligned} $$

By elimination of $\dot{\varphi }$ from three equations of Eq. (35), we obtain two equations of *u*_1_ and *u*_2_ as

36 $$ \begin{aligned} & - 8u_{1} + 8u_{1} u_{2} + 8u_{1}^{2} + 5a^{4} (1 - 18u_{1} - 2u_{2} + 73u_{1} u_{2} + 36u_{1}^{2} - 80u_{1}^{3} - 242u_{1}^{2} u_{2} - u_{2}^{2} - 212u_{1} u_{2}^{2} + 10u_{2}^{3} \\ & \quad + 135u_{1}^{4} + 450u_{1}^{3} u_{2} )/16 = 0, \\ & - 24u_{2} + 24u_{1} u_{2} + a^{4} (1 + 14u_{1} - 226u_{2} - 55u_{1}^{2} + 445u_{1} u_{2} + 90u_{1}^{3} + 990u_{2}^{2} - 1020u_{1}^{2} u_{2} - 3330u_{1} u_{2}^{2} \\ & \quad - 3160u_{2}^{3} - 75u_{1}^{4} + 1830u_{1}^{3} u_{2} )/16 = 0. \\ \end{aligned} $$

In general, *u*_1_ and *u*_2_ are small. So, it is possible to divide the first and second of Eq. (36) by 1 − *u*_1_ − *u*_2_ and then they become

37 $$ \begin{aligned} 384u_{1} & = a^{4} (15 - 225u_{1} - 15u_{2} + 825u_{1} u_{2} + 285u_{1}^{2} - 30u_{2}^{2} - 915u_{1}^{3} \\ & \quad - 2520u_{1}^{2} u_{2} - 2385u_{1} u_{2}^{2} + 120u_{2}^{3} ), \\ 384u_{2} & = a^{4} (1 + 15u_{1} - 225u_{2} + 235u_{1} u_{2} - 40u_{1}^{2} + 50u_{1}^{3} - 825u_{1}^{2} u_{2} \\ & \quad + 765u_{2}^{2} - 2330u_{1} u_{2}^{2} - 2395u_{2}^{3} ) = 0. \\ \end{aligned} $$

Therefore, by choosing *λ*_0_ = 16*a*^4^/(384 + 225*a*^4^), the power series solution of these equations are obtained as

38 $$ \begin{aligned} u_{1} & = \frac{{15\lambda_{0} }}{16} - \frac{{15\lambda_{0}^{2} }}{256} + \frac{{73095\lambda_{0}^{3} }}{4096} - \cdots , \\ u_{2} & = \frac{{\lambda_{0} }}{16} + \frac{{225\lambda_{0}^{2} }}{256} - \frac{{4935\lambda_{0}^{3} }}{4096} - \cdots . \\ \end{aligned} $$

Substituting the values of *u*_1_ and *u*_2_ into first equation of Eq. (35), and then simplifying, we obtain

39 $$ \dot{\varphi }_{3(q,Usual)}^{2} = 1 + \frac{{5a^{4} }}{8} - \frac{{65a^{8} }}{1536} + \frac{{3485a^{12} }}{131072} - \frac{{3953755a^{16} }}{226492416} + \cdots , $$

where, $\dot{\varphi }_{3(q,Usual)}^{2}$ denotes the third approximate frequency of the Eq. (7) by the usual HBM.

## Appendix 2

Substituting solution Eq. (4) (together with *u*_*j*_ = 0, *j* > 2) in Eq. (16) and equating the coefficient of cos $\varphi$, cos 3$\varphi$ and cos 5$\varphi$, the following nonlinear algebraic equations are obtained

40 $$ \begin{aligned} & \dot{\varphi }^{2} ( - 1 + u_{1} + u_{2} ) + 1 - u_{1} - u_{2} + 3a^{2} (1 - 2u_{1} - 3u_{2} + 3u_{1}^{2} + 6u_{1} u_{2} + 5u_{2}^{2} - 2u_{1}^{3} \\ & \quad - 4u_{1}^{2} u_{2} - 6u_{1} u_{2}^{2} - 3u_{2}^{3} + \cdots )/4 = 0, \\ & - 9u_{1} \dot{\varphi }^{2} + u_{1} + a^{2} (1 + 3u_{1} - 9u_{1}^{2} - 6u_{1} u_{2} - 3u_{2}^{2} + 8u_{1}^{3} - 25u_{1}^{4} + 6u_{1}^{2} u_{2} + 9u_{1} u_{2}^{2} + 2u_{2}^{3} + \cdots )/4 = 0, \\ & - 25u_{2} \dot{\varphi }^{2} + u_{2} + 3a^{2} (u_{1} + 2u_{2} - u_{1}^{2} - 6u_{1} u_{2} - 4u_{2}^{2} + 5u_{1}^{2} u_{2} + 5u_{1} u_{2}^{2} + 3u_{2}^{3} + \cdots )/4 = 0. \\ \end{aligned} $$

By elimination of $\dot{\varphi }$ from three equations of Eq. (40), we obtain two equations of *u*_1_ and *u*_2_ as

41 $$ \begin{aligned} & 8( - u_{1} + u_{1} u_{2} + u_{1}^{2} ) + a^{2} (1 - 25u_{1} - u_{2} + 72u_{1} u_{2} + 42u_{1}^{2} - 3u_{2}^{2} - 64u_{1}^{3} - 141u_{1}^{2} u_{2} - 117u_{1} u_{2}^{2} + 5u_{2}^{3} \\ & \quad + 46u_{1}^{4} + 94u_{1}^{3} u_{2} + \cdots )/4 = 0, \\ & 24( - u_{2} + u_{1} u_{2} + u_{2}^{2} ) + 3a^{2} (u_{1} - 23u_{2} - 2u_{1}^{2} + 41u_{1} u_{2} + u_{1}^{3} + 69u_{2}^{2} - 63u_{1}^{2} u_{2} - 3330u_{1} u_{2}^{2} \\ & \quad - 118u_{2}^{3} - 135u_{1} u_{2}^{2} + \cdots )/4 = 0. \\ \end{aligned} $$

Dividing the first and second of Eq. (41) by 1 − *u*_1_ − *u*_2_ and they are become

42 $$ \begin{aligned} u_{1} & = \mu_{1} (1 + 18u_{1}^{2} - 46u_{1}^{3} + 48u_{1} u_{2} - 3u_{2}^{2} - 75u_{1}^{2} u_{2} - 27u_{1}^{3} u_{2} - \cdots ), \\ u_{2} & = \mu (u - u^{2} + 19uv - 45u^{2} v + 46v^{2} - 70uv^{2} - \cdots ), \\ \end{aligned} $$

where *μ*_1_ = *a*^2^/(32 + 24*a*^2^) and *μ* = *a*^2^/(32 + 23*a*^2^).

The algebraic relation between *μ*_1_ and *μ* is

43 $$ \mu_{1} = \mu /(1 - \mu ) . $$

Expanding the right hand side of Eq. (43) in powers of *μ* and substituting this value of *μ*_1_ into the second equation of Eq. (42), it can be solved Eq. (42) in powers of *μ*_1_ as

44 $$ \begin{aligned} u_{1} & = \mu_{1} + 18\mu_{1}^{3} + 2\mu_{1}^{4} + 570\mu_{1}^{5} + 129\mu_{1}^{6} + 20642\mu_{1}^{7} + 6296\mu_{1}^{8} + \cdots , \\ u_{2} & = \mu_{1}^{2} + 37\mu_{1}^{4} + 4\mu_{1}^{5} + 1545\mu_{1}^{6} + 346\mu_{1}^{7} + 67039\mu_{1}^{8} + \cdots . \\ \end{aligned} $$

Substituting the values of *u*_1_ and *u*_2_ into first equation of Eq. (40), and then simplifying, we obtain

45 $$ \dot{\varphi }_{3(c,Usual)}^{2} = 1 + \frac{{3a^{2} }}{4} - \frac{{3a^{4} }}{128} + \frac{{9a^{6} }}{512} - \frac{{1773a^{8} }}{131072} + \frac{{5589a^{10} }}{524288} - \frac{{144008\tfrac{1}{2}a^{12} }}{ 1 6 7 7 7 2 1 6} + \cdots . $$

where, $\dot{\varphi }_{3(c,Usual)}^{2}$ denotes the third approximate frequency of the Eq. (16) by the usual HBM.

Dividing the first equation of Eq. (40) by 1 − *u*_1_ − *u*_2_, we obtain

46 $$ \dot{\varphi }^{2} = 1 + 3a^{2} (1 - u + 2u^{2} - 2v + 3uv + u^{2} v + 3v^{2} + u^{2} v^{2} + u^{2} v^{3} + 3u^{3} v^{3} \cdots )/4 $$

The third approximate solution measures better result when Eqs. (46) and (44) are truncated as

47 $$ \dot{\varphi }^{2} = 1 + 3a^{2} (1 - u_{1} + 2u_{1}^{2} + u_{1}^{3} - 2u_{2} + 2u_{1}^{2} u_{2} + 3u_{1}^{3} u_{2} - 2u_{2}^{2} - 2u_{1} u_{2}^{2} - 2u_{2}^{3} - 4u_{2} u_{2}^{3} + \cdots )/4. $$

and

48 $$ \begin{aligned} u_{1} & = \lambda_{1} (1 + 18u_{1}^{2} - 50u_{1}^{3} + 51\,u_{1} u_{2} - 58u_{1}^{3} u_{2} + 15u_{1}^{4} u_{2} + 54u_{1} u_{2}^{2} + 48u_{1}^{2} u_{2}^{2} + 50u_{1} u_{2}^{3} + \cdots ), \\ v_{1} & = \mu_{1} (u_{1} - u_{1}^{2} + 22u_{1} u_{2} - 50u_{1}^{2} u_{2} + 50u_{2}^{2} - 50u_{1}^{2} u_{2}^{2} + 50u_{2}^{3} + 50u_{1} u_{2}^{3} + 50u_{2}^{4} + 100u_{1} u_{2}^{4} + \cdots \,), \\ \end{aligned} $$

where *λ*_1_ and *μ*_1_ are given in Eqs. (42), (43).

The power series of Eq. (48) is obtained as

49 $$ \begin{aligned} u_{1} & = \mu_{1} + 18\mu_{1}^{3} + \mu_{1}^{4} + 620\mu_{1}^{5} + 7183\mu_{1}^{6} /25 + 657782\mu_{1}^{7} /25 + 819506\mu_{1}^{8} /25 + \cdots , \\ u_{2} & = \mu_{1}^{2} + 40\mu_{1}^{4} + 5\mu_{1}^{5} + 1874\mu_{1}^{6} + 22583\mu_{1}^{7} /25 + 2377007\mu_{1}^{8} /25 + \cdots . \\ \end{aligned} $$

## Appendix 3

Substituting the second approximate solution Eq. (4) in Eq. (23) and equating the coefficient of cos $\varphi$, cos 3$\varphi$, the following nonlinear algebraic equations are obtained

50 $$ \begin{aligned} & \dot{\varphi }^{2} \,\left( { - 4 + 4u_{1} + a^{2} (1 + 2u_{1} + 11u_{1}^{2} - 14u_{1}^{3} )} \right)/4 - 1 - u_{1} = 0, \\ & \dot{\varphi }^{2} \,\left( { - 36u_{1} - a^{2} (1 + 5u_{1} - 7u_{1}^{2} + 12u_{1}^{3} )} \right)/4 + u_{1} = 0. \\ \end{aligned} $$

By elimination of $\dot{\varphi }$ from three equations of Eq. (50), we obtain the equation of *u*_1_ as

51 $$ a^{2} + 32u_{1} - 5a^{2} u_{1} - 32u_{1}^{2} + 14a^{2} u_{1}^{2} - 8a^{2} u_{1}^{3} + \cdots = 0. $$

Now, the above equation can written as

52 $$ u_{1} = - (a^{2} - 5a^{2} u_{1} - 32u_{1}^{2} + 14a^{2} u_{1}^{2} - 8a^{2} u_{1}^{3} + \cdots )/32 . $$

It is clear that *u*_1_ is much smaller than 1 when *a* = O(1). In this case Eq. (52) can be solved in powers of *a*^2^ as *u*_1_ = *l*_1_*a*^2^ + *l*_2_*a*^4^ + *l*_3_*a*^6^ + ···, where the unknown coefficients, *l*_1_, *l*_2_, *l*_3_, …, to be determined.

Thus we have the solution of Eq. (52), as

53 $$ u_{1} = - \frac{{a^{2} }}{32} - \frac{{a^{4} }}{256} - \frac{{13a^{6} }}{163884} - \cdots . $$

Now substituting the value of *u*_1_ in the first equation Eq. (50), the approximate frequency (i.e., $\dot{\varphi }$) for small oscillation is obtained as

54 $$ \dot{\varphi }_{2(Existing)}^{2} = 1 + \frac{{a^{2} }}{4} + \frac{{5a^{4} }}{128} + \frac{{9a^{6} }}{2048} + \frac{{37a^{8} }}{ 6 5 5 3 6} + \cdots . $$

where $\dot{\varphi }_{2(Existing)}^{2}$ denotes the second approximate solution of Eq. (23) by the usual HBM.

In a similar way, the third approximate solution of Eq. (23) can be obtained as

55 $$ \begin{aligned} u_{1} & = - \frac{{a^{2} }}{32} - \frac{{a^{4} }}{256} + \frac{{a^{6} }}{16384} + \cdots , \\ u_{2} & = \frac{{7a^{4} }}{3072} + \frac{{31a^{6} }}{73728} + \cdots , \\ \end{aligned} $$

and

56 $$ \dot{\varphi }_{3(Existing)}^{2} = 1 + \frac{{a^{2} }}{4} + \frac{{5a^{4} }}{128} + \frac{{5a^{6} }}{1536} - \frac{{16\tfrac{5}{9}a^{6} }}{131072} - \cdots . $$

where $\dot{\varphi }_{3(Existing)}^{2}$ denotes the third approximate solution of Eq. (23) by the usual HBM.
